# Supplementary material for: Reversible long-range domain wall motion in an improper ferroelectric
Source: Nat Commun. 2025 Feb 19;16:1781. doi: 10.1038/s41467-025-57062-8 (PMC11840035; doi:10.1038/s41467-025-57062-8)
Supplement: Supplementary file 1 — Supplementary Information [file 41467_2025_57062_MOESM1_ESM.pdf]

Supplementary Information

Reversible long-range domain wall motion in an improper ferroelectric

M. Zahn[1,2], A.M. Müller[3], K. P. Kelley[4], S. M. Neumayer[4], S. V. Kalinin[5], I. Kézsmarki[2], M. Fiebig[3], Th. Lottermoser[3], N. Domingo[4], D. Meier[1,*], J. Schultheiß[1,6,*]

[1] Department of Materials Science and Engineering, Norwegian University of Science and Technology (NTNU), 7034, Trondheim, Norway

[2] Center for electronic correlation and magnetism, Institute of Physics, University of Augsburg, Germany

[3] Department of Materials, ETH Zurich, 8093 Zurich, Switzerland

[4] Center for Nanophase Materials Science, Oak Ridge National Laboratory, Oak Ridge, USA

[5] Department of Materials Science and Engineering, University of Tennessee, Knoxville, USA

[6] Department of Mechanical Engineering, University of Canterbury, 8140 Christchurch, New Zealand

1. Correction of BE-PFM signal for electro-mechanical contributions

In Figure S1a, the BE-PFM signal is displayed in a complex representation, visualizing the real and imaginary part[1] before the application of the bipolar triangular voltage signal sequence (indicated by 1) in Figure 1a. Reflecting the uniaxial ferroelectric nature of $ErMnO_3$, the data points are expected to cluster around two centers corresponding to the BE-PFM signal of the up- and down-polarized domains, respectively, forming a dumbbell-like closure. This general shape is observed for our data points. However, in addition a shift and a rotation of the closure with respect to the origin is observed, which can be attributed to electrostatic contributions. Figure S1b visualizes the BE-PFM signals in the complex plane for three positions: in +P and -P domains (orange and violet) and on a domain wall (green). As shown in Figure S1b, all three signals partially overlap. To correct for this effect, a parameterization is introduced as presented in Figure S1a. First, the two

cluster centers for a given electric field are determined using *k*-means clustering with the number of the clusters, $k = 2$, on the complex BE-PFM data set, resulting in cluster centers visualized by red and green dots in Figure S1a. The middle in between them is referred to as the center of the dumbbell and is highlighted with a black dot. This data treatment now allows to describe the data set via two vectors, one from the origin to the center point of the dumbbell, $\vec{o}$, and one from one cluster center to the other, referred to as connector, $\vec{c}$.

Based on the two vectors, the BE-PFM signal is corrected so that the two cluster centers are projected on the positive and negative *Re*-axis, respectively, by first subtracting $\vec{o}$, followed by a subsequent multiplication by the normalized complex conjugate of the connector, $\vec{c}^*/|c|$, to compensate the rotation. The procedure to determine the cluster centers and correct both described contributions is repeated for every electric field separately. In Figure S1c, the signal evolution is displayed after correcting for the same three data points as in Figure S1b, which can now be clearly identified as belonging to oppositely oriented ferroelectric domains. It is evident that the orange and violet data points correspond to the oppositely oriented ferroelectric domains, whereas the green data set corresponds to a ferroelectric domain wall.

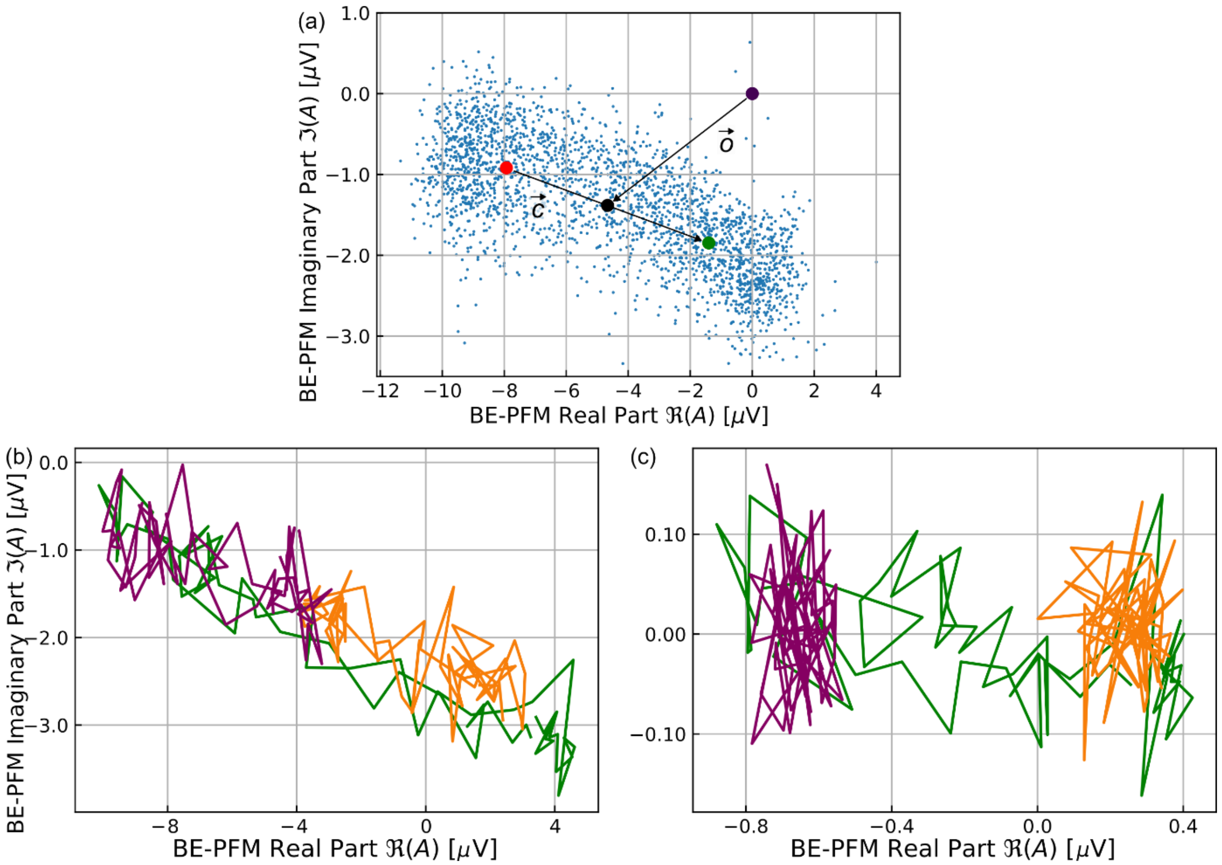

Figure S1: Separation of ferroelectric and electro-mechanical signals. a) The distribution of the BE-PFM signal in the complex plane before application of the bipolar triangular voltage signal sequence is of the expected dumbbell form. The dumbbell is displaced and rotated due to superimposed electromechanical contributions. To subtract for these electromechanical contributions, a parameterization with the vectors $\vec{o}$ (origin) and $\vec{c}$ (connector) is introduced. b) Evolution of the raw BE-PFM signal in the complex before compensating for the electro-mechanical contributions. Orange and violet data correspond to differently oriented domains, while green data points correspond to a domain wall. c) After compensating the electro-mechanical contributions, this becomes clear.

Figure S2a gives an overview of the evolution of the complex representation of the BE-PFM signal over the entire applied bipolar voltage sequence (Figure 1a). To analyze the evolution of the data systematically, we focus on the parameterization vectors introduced in Figure S1a. The trajectory of the center of the dumbbell captured by the origin vector, $\vec{o}$, is shown in Figure S2b. A clear voltage dependence of the origin vector is found, as outlined by the characteristic applied electric fields, 1-4. As explained in ref. [2], the difference in work function between tip and sample leads to a contact potential and an

additional contribution to the captured signal, even in the absence of an external electric field. For our data set, this difference manifests in the displacement of the dumbbell from the origin of the complex plane. Since the BE-PFM measurement is performed in the absence of an electric field (see method section), a variation of the origin vector with electric field, as we observe in Figure S2b, is most likely a transient effect. This is supported by the fact that the found linear variation of the origin vector with the electric field is consistent with the theory introduced in ref. [2].

Next, the trajectory of the connection vector, $\vec{c}$, is displayed as amplitude and phase in Figure S2c. The phase is constant and independent of the applied electric field and can be calibrated to zero. The amplitude displays two local minima and maxima within the bipolar voltage profile sequence, indicating that the underlying effect couples quadratically to the applied electric field. According to the theory of electrostatic force microscopy,[3] free charge carriers, that screen the electric field, might accumulate or disperse for both direction of the field equally.

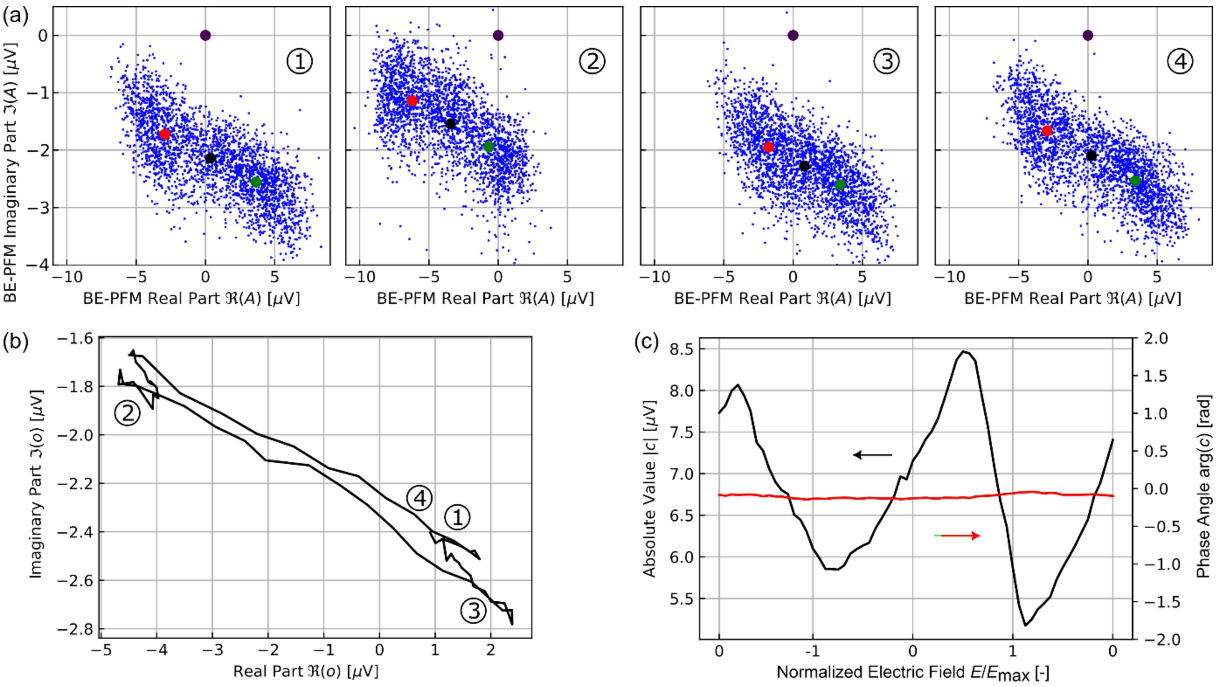

Figure S2: Separation of ferroelectric switching and electro-mechanical signals over the bipolar voltage profile. a) Evolution of the BE-PFM signal in the complex representation over the entire bipolar voltage signal sequence. The respective cluster centers described in Figure S2a are displayed. The numbers 1-4 are introduced in Figure 1a. b) Evolution of the extracted complex origin vector, $\vec{o}$, over the entire bipolar voltage signal sequence (Figure 1a). The applied voltage states are indicated by the numbers 1-

4. c) Evolution of the connection vector, $\vec{c}$, as amplitude and phase as a function of the bipolar voltage signal sequence.

2. BE-PFM data showing ferroelectric order before voltage sequence application

Figure S3 shows the phase and amplitude to the BE-PFM signal. Both domains are separated by a π rad BE-PFM phase difference with black domains pointing inwards and white domains outwards. The BE-PFM amplitude is equal in both domains, indicating a non-zero piezoelectric response within the domains, while a lower contribution is observed at the ferroelectric domain walls, indicating that the piezoelectric contribution at the ferroelectric domain walls vanishes[4].

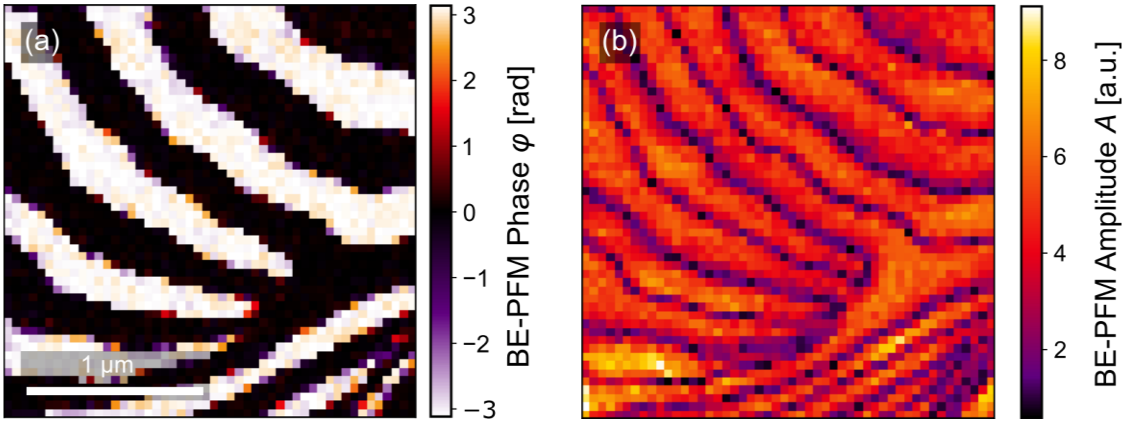

Figure S3: BE-PFM data showing the domain structure before the application of bipolar triangular voltage signal sequence. Corrected phase and amplitude data are displayed in a) and b), respectively. The phase data verifying the close to a π rad phase shift between the oppositely oriented domains. The amplitude data displays a clear minimum on every domain wall independent of its orientation, indicating a vanishing of the piezoresponse at the domain wall.

3. Evolution of the domain structure recorded at a second position

BE-PFM data has been recorded at different positions with the same bipolar electric field sequence. In addition to the data presented in the main text, a second data set recorded at a different position on an out-of-plane oriented grain is presented in Figure 1a. Similarly to Figure 1b-d, a coherent contraction and expansion of domains is observed. Again, the domain wall displacement is most pronounced away from the structural vortex lines, which can be for example observed in the stripe-like domains in the upper left corner of the BE-PFM image sequence.

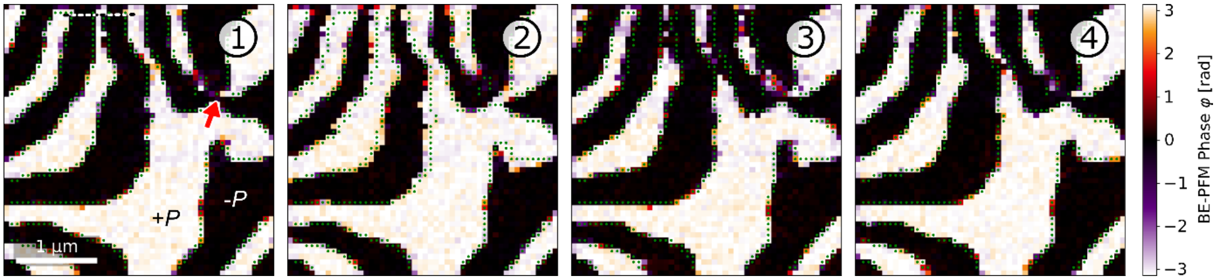

Figure S4: Evolution of the BE-PFM phase recorded at a second position on the same material under a bipolar voltage sequence. The numbers 1-4 correspond to applied electric fields as introduced in Figure 1a. White areas represent upwards-oriented domains, +*P*, and black areas represent downwards-oriented, -*P*, domains. The red arrow indicates the position of the intersection of a vortex line with the surface. Green dots highlight the initial positions of the ferroelectric domain walls, showing their evolution with applied voltage. Similar to Figure 1, near the structural vortex lines, we do not resolve domain wall movements. A notable reversibility of the domain structure is observed, for example in the stripe-like domains in the upper left corner. A movie showing the electric-field dependent evolution of the BE-PFM phase and amplitude is provided in Supplementary Movie 3. The time-dependent BE-PFM phase (after the application of the DC pulses) is extracted along the dashed white line and displayed in Figure S5.

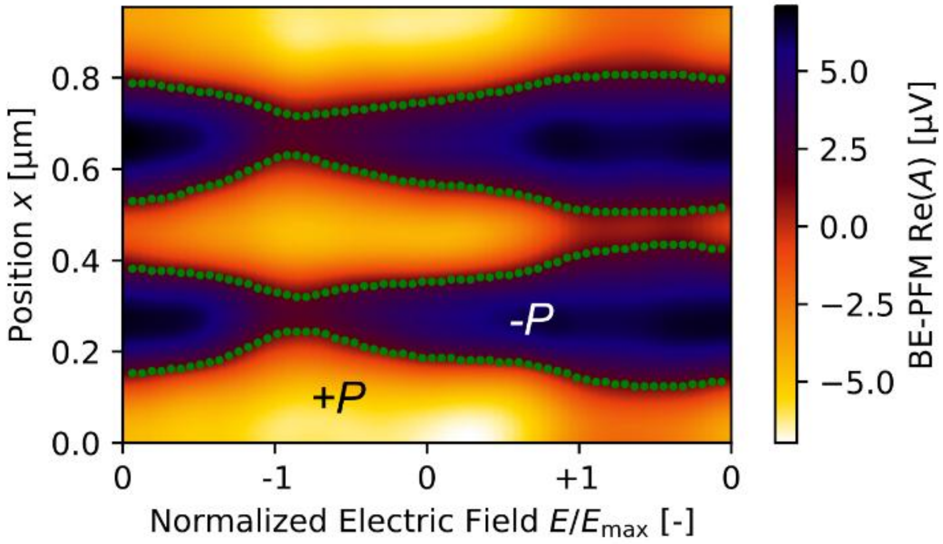

Figure S5: Influence of an electric field on ferroelectric domains observed for a pair of ferroelectric domain walls. Voltage-dependent experimental data are shown as BE-PFM phase extracted along the dotted line in Fig. S4. The green dots serve as a visual guide, indicating the electric-field-dependent positions of the domain wall, determined through thresholding. The walls traverse distances up to 150 ± 25 nm, returning to their original positions, moving between maximum positive and negative voltages. No crosstalk is observed between the two neighboring domains that would influence the reversible domain wall movement.

4. Stability of domain patterns in phase field simulations

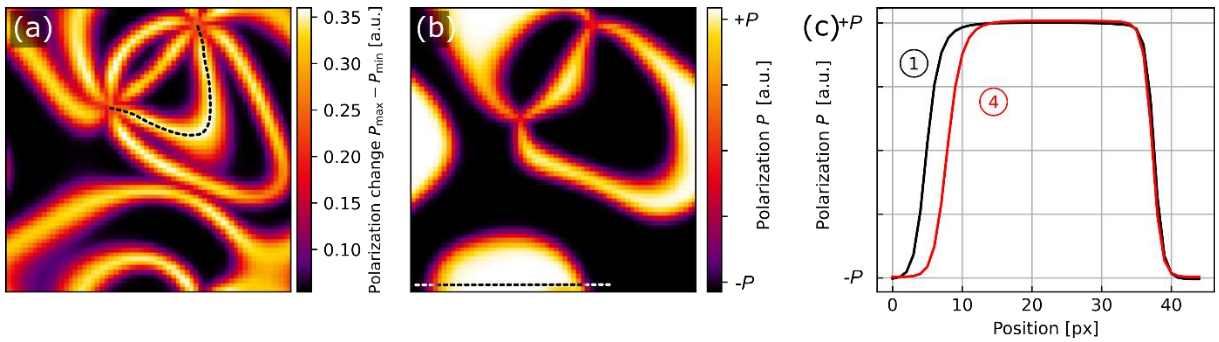

Figure S6: Domain pattern in phase field simulation. a) Depicted is the maximum change in polarization for each spatial point over the entire electric field sweep cycle. Accordingly, bright points are those switching during the cycle, respectively a domain wall passes these points. An exemplary domain wall with fixed ends in two different vertices is highlighted by a dashed line. Largest domain wall motion amplitude is observed in the middle of the line while the motion decreases in areas of high domain wall density and vanishes at the vertices. b) Exemplary domain structure in the phase field simulation before applying the triangular electric field sweep cycle. For comparison of initial and final state, the polarization along the dashed line is extracted. c) Comparison of the ferroelectric polarization before (state 1) and after the field sweep (state 4).

Supplementary references

[1] T. Jungk, A. Hoffmann, E. Soergel, *J. Microsc.* **2007**, *227*, 72.
[2] N. Balke, S. Jesse, P. Yu, B. Carmichael, S. V. Kalinin, A. Tselev, *Nanotechnology* **2016**, *27*, 425707.
[3] J. Schaab, A. Cano, M. Lilienblum, Z. Yan, E. Bourret, D. Meier, R. Ramesh, M. Fiebig, *Adv. Electron. Mater.* **2016**, *2*, 1500195.
[4] E. Soergel, *J. Phys. D Appl. Phys.* **2011**, *44*, 464003.
