# Supplementary material for: Reversible long-range domain wall motion in an improper ferroelectric
Source: Nat Commun. 2025 Feb 19;16:1781. doi: 10.1038/s41467-025-57062-8 (PMC11840035; doi:10.1038/s41467-025-57062-8)
Supplement: Supplementary file 2 — Description Of Additional Supplementary File [file 41467_2025_57062_MOESM2_ESM.pdf]

Description of Additional supplementary files

Supplementary Movie 1: BE-PFM amplitude and phase images showing the evolution of the ferroelectric domain structure during the electric field application, following the bipolar triangular voltage signal sequence of Figure 1a.

Supplementary Movie 2: Simulations showing polarization images obtained from phase field simulations. The data documents the evolution of the ferroelectric domain structure during the electric field application.

Supplementary Movie 3: BE-PFM amplitude and phase evolution analog to Supplementary Movie 1 for the position evaluated in Figure S4.
