# Supplementary figures and images for: Reversible long-range domain wall motion in an improper ferroelectric
Source: Nat Commun. 2025 Feb 19;16:1781. doi: 10.1038/s41467-025-57062-8 (PMC11840035; doi:10.1038/s41467-025-57062-8)

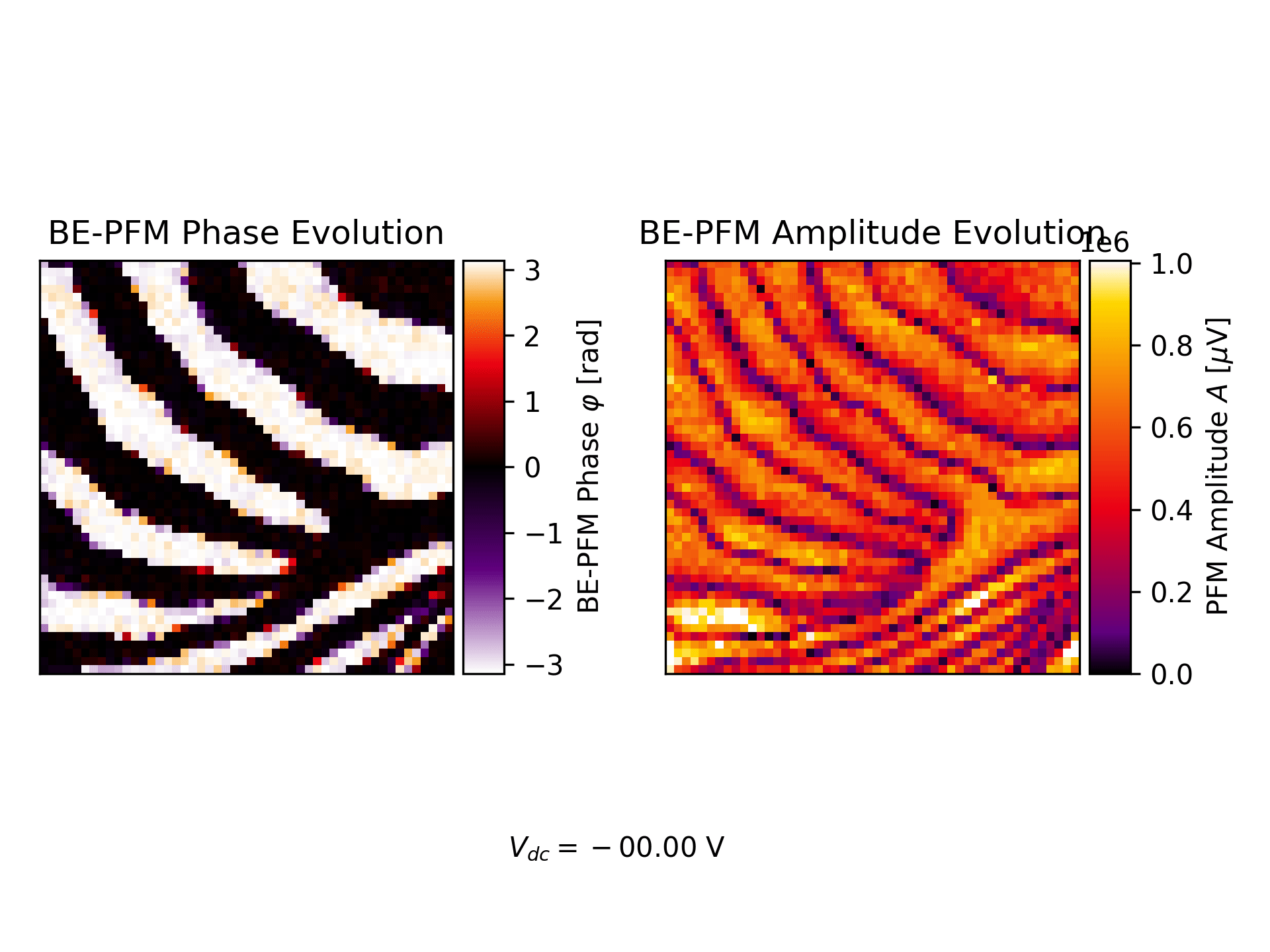

Supplement: Supplementary file 3 — Supplemenatry Movie 1 [file 41467_2025_57062_MOESM3_ESM.gif]

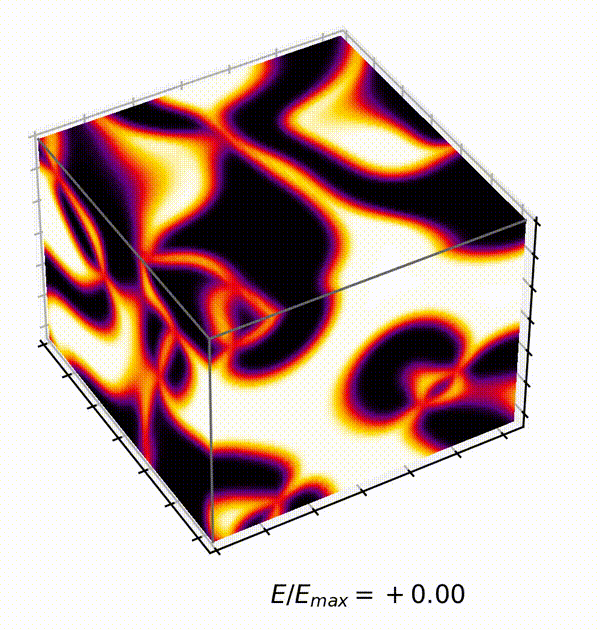

Supplement: Supplementary file 4 — Supplemenatry Movie 2 [file 41467_2025_57062_MOESM4_ESM.gif]

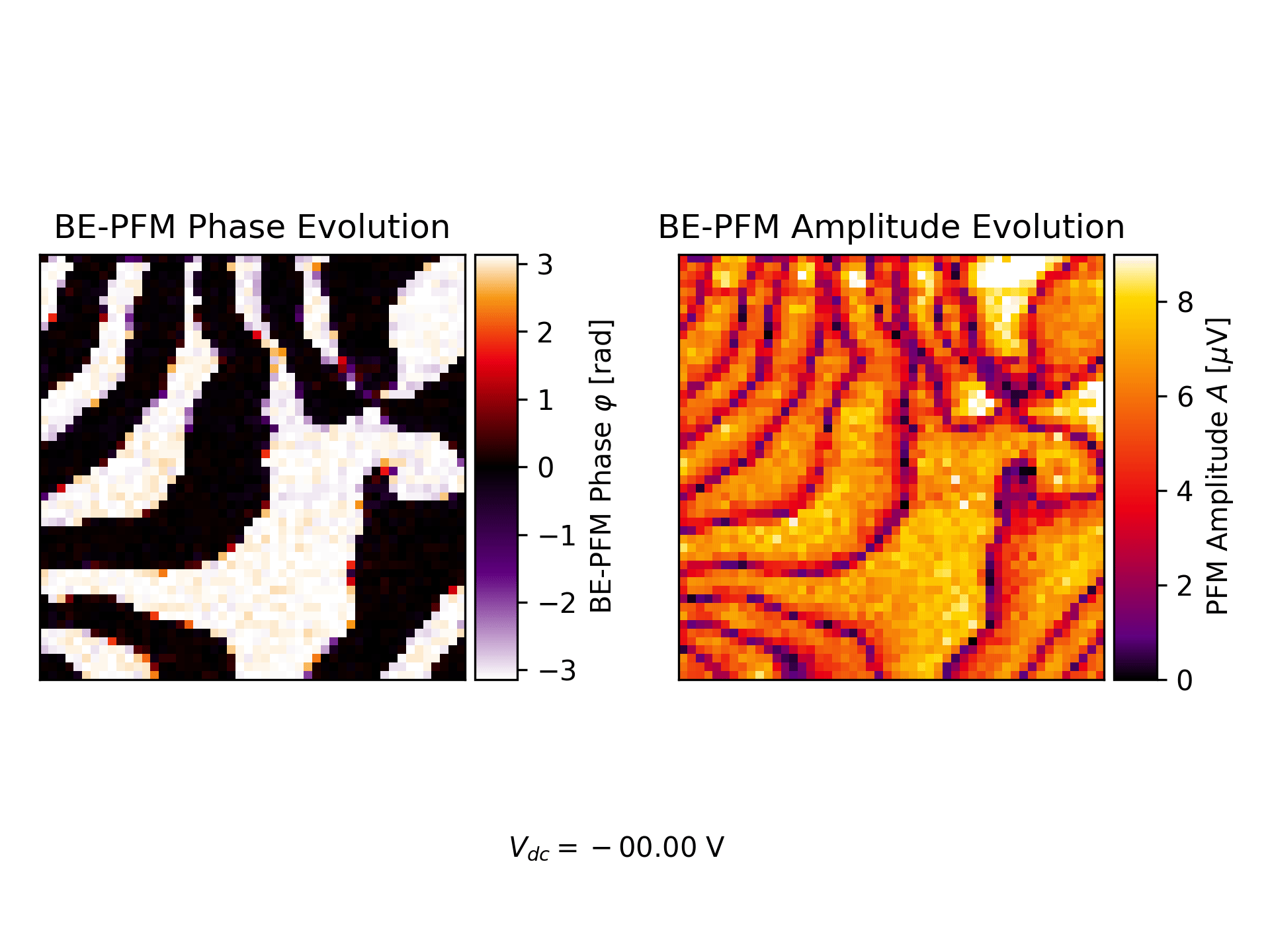

Supplement: Supplementary file 5 — Supplemenatry Movie 3 [file 41467_2025_57062_MOESM5_ESM.gif]
